# A Capacitive Displacement Sensing Technique for Early Detection of Unbalanced Loads in a Washing Machine

**DOI:** 10.3390/s91209559

**Published:** 2009-11-30

**Authors:** Melur K. Ramasubramanian, Karthik Tiruthani

**Affiliations:** 1 Mechanical and Aerospace Engineering, North Carolina State University, Broughton Hall 1217, Box 7910, NCSU Campus, Raleigh, NC 27695, USA; 2 Mechanical and Aerospace Engineering, North Carolina State University, Box 7910, NCSU Campus, Raleigh, NC 27695, USA; E-Mail: krtiruth@ncsu.edu

**Keywords:** capacitive-displacement-sensor, horizontal-axis-washing-machine, vibration-detection

## Abstract

Horizontal axis washing machines are water and energy efficient and becoming popular in the USA. Unlike a vertical axis washer, these do not have an agitator and depend solely on tumbling for the agitation of laundry during the wash cycle. However, due to the constant shifting of laundry during washing, the load distribution is often unbalanced during the high speed spin cycle. We present a displacement-based sensing method to detect unbalance early while the spin rate (rpm) is well below the resonance frequency so that corrective actions may be taken prior to the high speed spin cycle. Experimental and analytical characterizations of the sensor configuration are presented. Results show that the displacement sensor is more appropriate than an accelerometer for this application and offer the potential for a simple, reliable, low cost detection of unbalance.

## 1. Introduction

**Figure 1.**
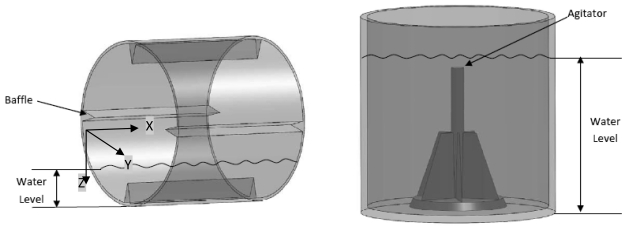
Horizontal and vertical axis washing machine configurations.

By using tumble-action for washing, the horizontal axis washing machine uses about half the water required for the average vertical axis washer. A schematic of both types of machines are shown in Figure 1. Further, high speed spin cycle in a horizontal washer results in an additional 10% moisture removal, which translates to lower energy usage in heated drying.

Horizontal axis washing machines also have innate unbalance problems associated with their design which can typically create a force in excess of 20 kN during the spin cycle [1]. Traditionally to counteract these forces a large mass in the form of concrete or cast iron block is added to the system. This added mass can be as much as 60% of the washer's total mass. Furthermore the addition of this large mass to the system results in over-design of structural components. The vertical axis washer can have unbalances of the same order of magnitude as the horizontal axis washer but the forces are primarily in the plane of motion [2], causing no major harm.

The significance of this problem in a horizontal axis machine increases as the manufacturers try to lower the weight of the washer and increase the spin cycle speeds. Dynamic unbalances and changes in center of gravity due to change in operating conditions such as placement of the laundry load by the user cannot be accounted for ahead of time [3]. Although the user behavior is not controllable, detection of unbalance in washing machines and its correction is essential to prevent the washer from spinning up to its full speed with unbalanced load, through resonance, causing excessive noise or even some structural damage to the washing machine. Solving this problem is very important especially in machines designed for residential use.

The objective of this paper is to describe a simple, cost effective sensing method to detect the possibility of severe vibration at high speed, well before it is perceivable by the user. The paper describes the measurement principle, sensor design, and results from experiments with an actual horizontal washing machine instrumented with this sensor. The sensor will be useful in providing reliable input to control algorithms designed to correct the anticipated vibration at high speed before it occurs.

## 2. Review of Unbalance Detection and Control Methods

One approach to avoid the effects of unbalance is to develop an automated way of balancing the load through mechanical devices and structures around the drum, for example, balance rings or fluids flowing into pathways around the drum. The theory behind this approach is discussed in depth in [4], and different forms of implementation are discussed in [5-8]. Even though these methods of using inertial forces for balancing are fairly successful they are usually complicated and expensive to implement. There have been several techniques described in patent literature that describe a decoupled measure and correct approach. Use of motor torque ripple, acceleration, velocity ripple, speed profile, and magneto-rheological dampers to measure and correct vibration due to unbalance loads are discussed in [9-14]. While some of the mechanical self-balancing methods, or methods that use an array of accelerometers, and sophisticated magneto-rheological (MR) dampers, are shown to work, the complexity of mechanisms involved, potential maintenance issues, and most importantly, the cost of implementation of these systems, prevent them from becoming practical.

## 3. Experiments with an Accelerometer

The objective of this work is to design a low cost unbalance detection method to detect unbalance at spin speeds (rpm) well below the resonance frequency of the system. Initially testing was performed at 400 rpm which was the lowest spin cycle speed we could drive the standard washing machine used in this study. The measurements were recorded with through experiments with a dual axis accelerometer (ADXL 202, Analog Devices Inc.) [15] For each axis, the value of the duty cycle at zero acceleration was recorded experimentally, since it can be different from 50%, the ideal central value, due to individual sensor construction variability. Table 1 summarizes the results from the tests. The quantities measured in Table 1 are the accelerations in the front-back axis (X-axis, ref. Figure 1) of the machine and side-side axis (Y-axis, ref. Figure 1) of the drum of the washing machine. The first column shows the unbalanced loading condition simulated in the experiments using disk magnets of known weight placed at desired locations inside the drum.

**Figure 2.**
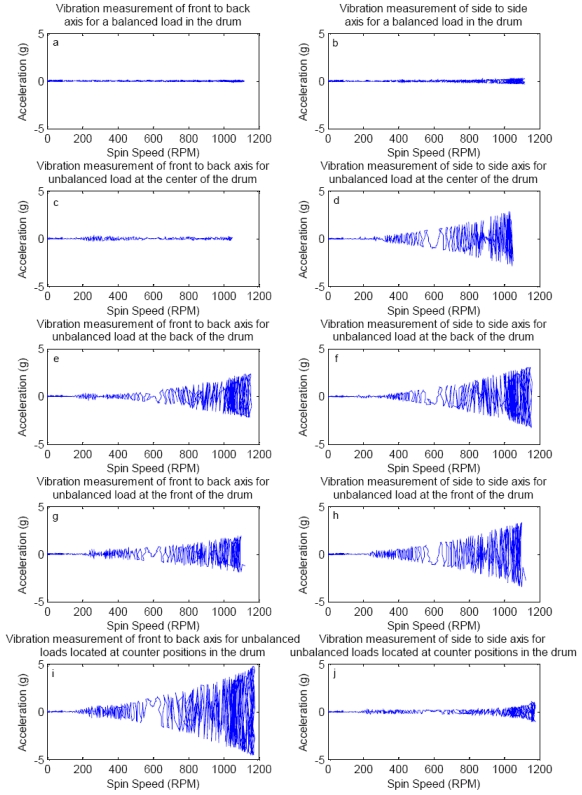
Acceleration measurements of fixed unbalance at some different locations in the drum.

From Table 1 we see that some side-to-side vibration exists even when there is no unbalanced load in the drum, whereas there is no front-to-back vibration. The side-to-side vibration is attributed to the characteristics of the machine suspension system. Figure 2 shows continuous acceleration measurements during a speed sweep test from zero to over 1,000 rpm, with unbalance load of 0.5 kg, placed at various locations in the drum.

From the results of test shown in Figure 2 it is observed that the resonance occurs around 250 rpm (ref. Figure 2e, for example), as indicated by a local peak in the front-to-back acceleration measurement, which is not detected in the side-to-side axis (ref. Figure 2f, for example). We also see that unbalance detection using the accelerometer starts near 200 rpm and front-to-back axis measurement is more sensitive at lower rpm. We also see that front-to-back acceleration measurement works better for detection in case of unbalanced loads in counter positions which was observed to be the worst case. Our objective is to detect unbalances, if present, at or below 100 rpm which gives a reasonable amount of time (about 3-4 seconds if the drum is ramping at 40 rpm/sec) before the drum reaches a resonant frequency to take any corrective action necessary and ensure that we do not spin an unbalanced drum through resonance. This is not possible with standard commercial accelerometers, as seen in the initial results shown in Figure 2. Thus it may be necessary to use a more sensitive method of detection of vibration at low speeds to detect unbalance. Further, it can be observed that the vibration measurement along the front to back axis is more sensitive to detect unbalance than side to side movement for any of the unbalance cases studied.

If displacement (*D*) is sinusoidal (*sin ωt*) then the velocity (*V*) is proportional to *ω*, and acceleration (*A*) is proportional to *ω^2^ i.e., V* = *ωD and A* = *ω^2^D*, where *ω* = *2πf* and *f* is frequency of vibration. So at lower frequencies displacement D is larger than acceleration A and thus easier to measure. Since our desired detection speed is 100 rpm, it is desirable to detect vibration by making displacement measurements instead of acceleration.

**Figure 3.**
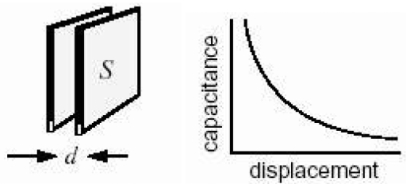
Parallel plate capacitance configuration and capacitance *vs.* displacement relation.

One of the simplest ways to measure displacement is to use a parallel plate capacitor, as shown in Figure 3. Capacitive sensing offers many advantages. The capacitive sensing system responds to average displacement of a large area of a moving electrode, thereby providing a robust measurement in practice. The sensing method also has a high signal to noise ratio. An important feature of capacitive sensing for this application is that it is less sensitive to lateral movement or misalignment of the plates, especially when the area S is large and thus it can respond to motion along the axis perpendicular to the plates and minimize the effect of slight motion parallel to the plates.

**Figure 4.**
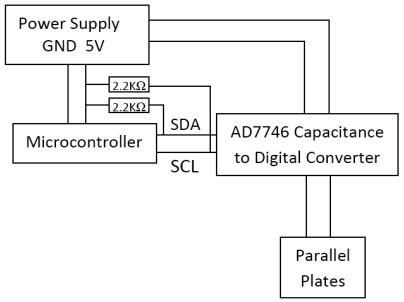
Circuit diagram showing measurement of displacement.

**Figure 5.**
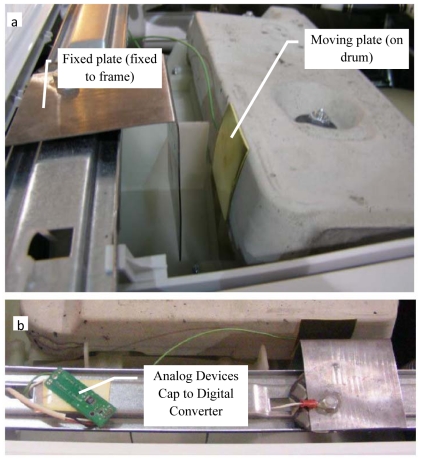
(a) Parallel plate configuration setup for displacement measurement on washing machine. (b) Capacitance to digital converter (Analog Devices, AD7746).

The circuit diagram used to measure this capacitance is shown in Figure 4. The data is read using a microcontroller through the I2C communication protocol which uses two lines, SCL (Serial Clock), and SDA (Serial Data), respectively. The hardware setup for the sensing system is shown in Figure 5. Figure 5(a) shows the parallel plate arrangement photographed from the side, an arrangement where the capacitance between the parallel plates changes as the drum moves. The area of the plates is 2580.6 mm^2^ (4 sq. in.) and they are placed at a nominal distance of 23.5 mm from each other. The change in capacitance is a direct indicator of the displacement which is related to unbalanced load in the drum. The plates are connected to a capacitance to digital converter (Analog Devices, AD7746) which outputs a digital value, shown in Figure 5(b).

**Figure 6.**
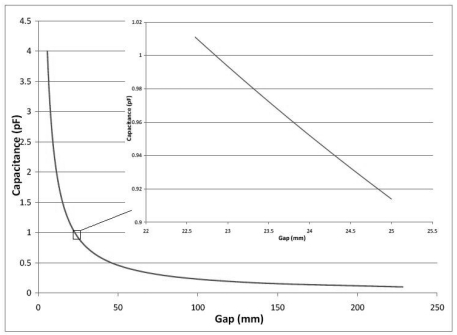
Capacitance *vs.* deflection relation for the sensor setup.

Figure 6 shows a plot of theoretical capacitance *vs.* distance between the plates, called the gap, for the setup used. The maximum displacement of the moving plate relative to the fixed electrode plate exceeds 20 mm when the machine is ramped up to 1,200 rpm, the full spin cycle speed, when there is an unbalance. We see that the gap to capacitance relationship is nearly linear and most sensitive for gaps below 20 mm but placing the plates at less than 20 mm away would result in the plates coming in contact during the operation of the machine. Hence, it was decided to place the electrodes as far apart as possible in this nearly linear region, *i.e.*, at 23.5 mm since the maximum range of motion during the low speed detection phase is ±1.5 mm from the mean gap. The capacitance versus gap for this range of motion is shown in the inset in Figure 6, and the behavior is well approximated to be linear.

## 4. Modeling

There have been many attempts at modeling the suspension system of a washing machine. A six degree of freedom motion model of the washing machine drum using a three dimensional rigid body model for the drum considering displacements and rotations about all three axes, when subjected to an unbalanced load is described in [16,17]. A three dimensional dynamic model of a horizontal-axis portable washing machine is described in [18]. The model is used to predict a critical speed for which rotational slip of the cabinet is impending for some unbalanced load. A two-dimensional rigid body model for a washing machine with and without the use of balance rings, and a three dimensional model for the washing machine without balance rings or unbalanced mass are described in [19].

To implement a three-dimensional model for real-time control will involve interfacing multiple sensors increasing the cost for both computing and sensing, and render the method impractical. A simple model that can accurately predict the behavior of the suspension system along one axis is desirable for microcontroller implementation is desirable.

**Figure 7.**
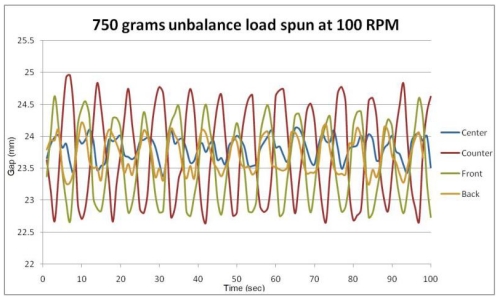
Displacement *vs.* Time plot for test carried out at 100 rpm with 750 g unbalanced load.

Results from tests performed with 0.75 kg unbalanced loads located at front, center, back and counter positions in the drum (the unbalanced loads are precisely simulated using magnets placed at various positions in the drum) are shown in Figure 7. It is observed that the frequency of vibration remains the same for all types of unbalanced loads, but the amplitude changes with the type of unbalance from being lowest for center to highest for counter positions of unbalance load. The displacement data looks like the output of a spring-mass system under harmonic excitation. Hence, we developed a simplified spring damper model to describe the behavior of the drum along the front to back axis subjected to harmonic excitation.

## 5. Simplified Model and Analysis

**Figure 8.**
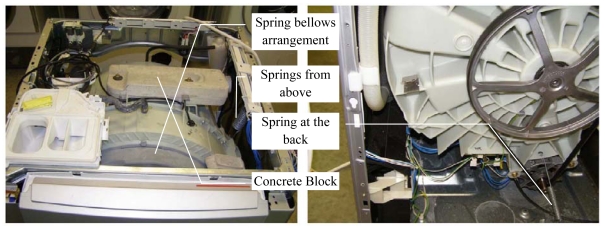
Washing machine drum's suspension.

The suspension system for the drum is as shown below in Figure 8. The drum is suspended by three springs, two from above and one from the back. There are also two dampers not seen in figure which are part of the suspension system. There is also a spring bellow arrangement that allows the drum to move freely while keeping the door stationary, and providing the seal for water.

**Figure 9.**
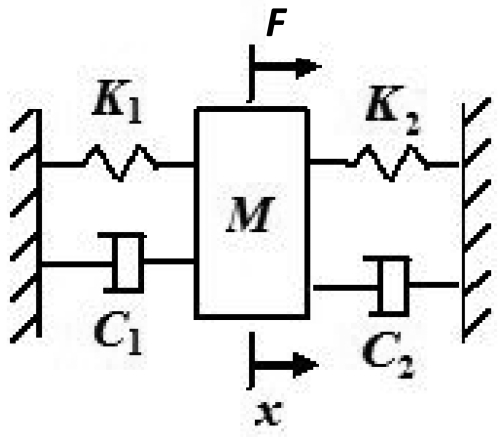
One dimensional spring mass system.

We approximate the model of the suspension system by a simple one dimensional spring mass system by considering equivalent spring stiffness, damping constants and forces in that direction and determine how closely the simplified model predicts the data. The system shown in Figure 9 is a representation of the washing machine drum suspension and *K_1_* represents the stiffness component in the front to back direction of the machine (X-axis) of the spring at the back, and *K_2_* represents the stiffness component in the x-axis of the combined effect of the two springs at the top. The effective damping is the combined effect of both the dampers *C_1_* and *C_2_* in the X- direction. The force acting on the system is a harmonic excitation due to the unbalanced mass being rotated at 100 rpm and we consider the component along the x-axis. Mass of the system m is the combined mass of the drum including the load and the concrete block (shown in Figure 8) on the top of the drum and the unbalanced mass.

The equation of motion for the above system given in [20] is

(1) $$m\ddot{x}+c\ddot{x}+kx=F$$

Where:

*m* is the combined mass of the system including the unbalanced mass
*c* is the effective damping constant *i.e., C_1_* + *C_2_*
*k* is the effective spring constant *i.e., K*_1_ + *K*_1_
*F* is the force due to unbalanced mass *i.e., u_m_e*ω^2^*sin*ω*t* or ƒ_0_*sin*ω*t* where ƒ_0_ = *u_m_e*ω^2^.

For our system, the parameters are *ω* = 10.472 rad/s (100 rpm), *m* = 20 Kg, *C* = 90 kgf-s/m and *K* = 8,300 N/m. The values of C and K were provided by the manufacturer. The top springs make an angle of 10°, the back spring makes an angle of 45°, and the dampers make an angle of 64° with the vertical axis. So the Effective Damping for the system is c = 2 Cg cos64 = 774.0758 and the effective Stiffness. k = 2K sin10 + K cos45 = 8,751.5 N/m. For this problem the value of unbalanced mass is *u_m_* = 0.75 Kg, and the distance from center of mass of unbalance (eccentricity) is estimated as *e*=0.1143 m (*i.e., r*/2) for center unbalance, *e* = 0.4572 m (*i.e.*, $\sqrt{{\left(r/2\right)}^{2}+{\left(0.691d\right)}^{2}}$ (where *r* is radius and d is depth of the drum, *r* = 228.6 mm and *d* = 640 mm) for front unbalance, *e*=0.2286 m (*i.e.*, $\sqrt{{\left(r/2\right)}^{2}+{\left(0.309d\right)}^{2}}$) for back unbalance and *e* = 0.5495 m for counter unbalance.

Solving the equation (1) and applying the parameter values for the specific system, we have: $\text{Natural Frequency}=\omega_{n}=\sqrt{\frac{k}{m}}=20.8193\;\text{rad}/s$, $\text{Damping Ratio}=\zeta =\frac{c}{2m\omega_{n}}=0.9251$, $\text{Frequency Ratio}=r=\frac{\omega }{\omega_{n}}=0.5006$, $\text{Damped Natural Frequency}=\omega_{d}=\sqrt{1-\zeta^{2}}\omega_{n}=7.9424\;\text{rad}/s$

The solution to this system given in [20] is:

(2) $$x(t)=X\sin\;(\omega_{d}t-\varphi )$$

Where, *Steady State Amplitude*, $X=\frac{f_{0}}{m\omega_{n}^{2}}\frac{1}{\sqrt{{\left(1-r\right)}^{2}+{\left(2\zeta r\right)}^{2}}}$ and *Phase Difference* $\varphi =\tan^{-1}\left(\frac{2\zeta r}{1-r^{2}}\right)$.

## 6. Results and Discussion

**Figure 10.**
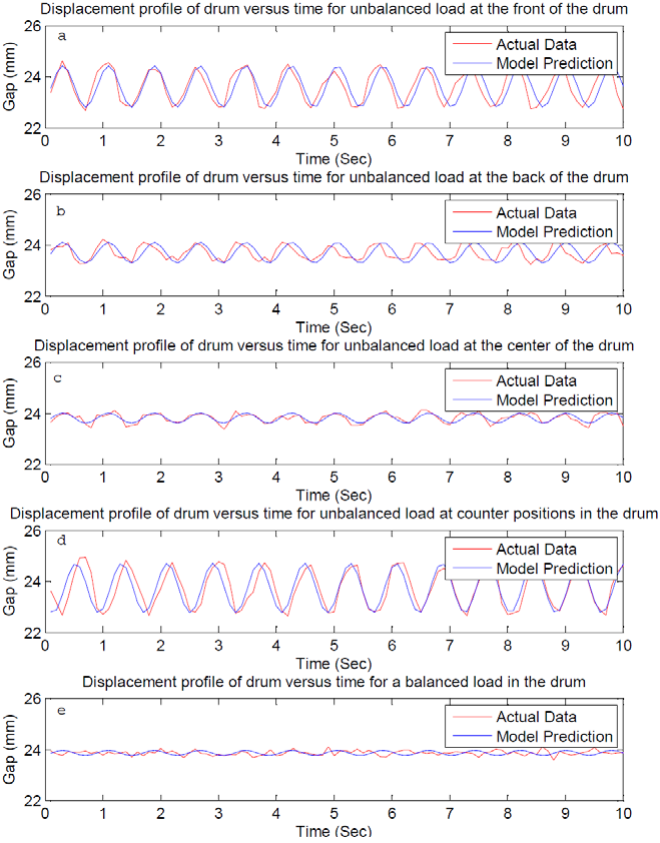
Displacement profile of drum versus time for unbalanced load at different locations.

Figure 10 shows the measurement of gap between the plates versus time for an unbalanced load (0.75 kg) located at different locations compared with the one-dimensional model prediction (Equation 2).

It is seen that in all cases, the agreement between model predictions and the experimental results is good. Further, the displacement amplitude depends on the location of the unbalance load. The unbalances located at front and counter positions in the drum show a significantly large displacement relative to unbalances located in the back and center of the drum, with the smallest unbalance magnitude for center unbalance. The measured frequency and the damped natural frequency are also in good agreement. All displacements are measured at 100 rpm, and the sensor is able to detect unbalance and the effect of location of unbalance. In Figure 10e, the measured displacement is not sinusoidal, and seems to have several high frequency components however, the model predicts small sinusoidal amplitude due to free vibration of the system, where the amplitude is of the same order of magnitude as the amplitude of the noise signal; it is a measurement of system noise and not significant.

Using the proposed method of displacement measurement and the one dimensional model, measurements of displacement can be input into the model and an equivalent unbalance load can be estimated for feeding into suitable corrective algorithm in real-time. For instance, if we establish that 0.75 kg in the front is the tolerable limit, then for any displacement value below the value predicted by the model, no corrective action will be performed by the machine. Only one sensor, and hence one input to the microcontroller is needed, reducing I/O requirements and cost. All this makes this method very attractive for the detection of unbalanced in a horizontal axis washing machine. This model can be easily modified for any other suspension configuration by changing the stiffness and damping parameters.

## 7. Conclusions

We conclude that a simple parallel plate capacitive sensor with a capacitance measurement circuit works well for the early detection of vibration in horizontal axis washing machines well below resonance. This is not possible with an accelerometer based system at comparable cost. A one dimensional model is shown to be satisfactory to predict the presence of unbalance satisfactorily. Using the proposed method of displacement measurement and the one dimensional model, measurements of displacement can be converted to an equivalent unbalance load in real-time and used to guide corrective measures. The model estimates the magnitude of unbalance and not its location. For practical implementation, exact location of unbalance may not be necessary. The corrective action generally consists of shuffling the load by back and forth through a rocking movement of the drum, regardless of the location of unbalance. This model can be easily modified for any other suspension by changing the stiffness and damping parameter values and the machine configuration.

## Figures and Tables

**Table 1.** Unbalance detection tests carried out at 400 rpm.

Loading Conditions	Acceleration –Side-to-side vibration axis (Y-axis) %Duty Cycle (Acceleration in g)	Acceleration –front-to-back vibration axis (X-axis) %Duty Cycle (Acceleration in g)
No Load	46.6%–48.9% (−0.08 g to 0.14 g)	∼46.5% (∼0 g)
250 grams load placed at the center of the drum	44%–50% (−0.24 g to 0.24 g)	45.5%-47.5% (−0.08 g to 0.08 g)
250 grams load placed at door of the drum	44.5%–51.3% (−0.21 g to 0.32 g)	42.6%-50% (−0.32 g to 0.28 g)
250 grams load placed at the back of the drum	45%–49.5% (−0.16 g to 0.2 g)	44%-49% (−0.2 g to 0.2 g)
250 grams loads diagonally placed in radially opposite locations in the front and back planes of drum	44%–50% (−0.24 g to 0.24 g)	40%-52% (−0.52 g to 0.44 g)
Zero G values given in percentage duty cycle are Y-Axis [47.1%] and X-Axis [46.5%]		
